# MOREshiny: a user-friendly application for the inference of phenotype-specific multi-omic regulatory networks

**DOI:** 10.1093/bioadv/vbag175

**Published:** 2026-06-18

**Authors:** Maider Aguerralde-Martin, Roxana Andreea Moldovan, María Verdú, Sonia Tarazona

**Affiliations:** Department of Applied Statistics, Operations Research and Quality, Universitat Politècnica de València, Valencia 46022, Spain; Department of Applied Statistics, Operations Research and Quality, Universitat Politècnica de València, Valencia 46022, Spain; Institute for Integrative Systems Biology, Spanish National Research Council, Catedràtic Agustín Escardino Benlloch, Paterna 46980, Spain; Department of Applied Statistics, Operations Research and Quality, Universitat Politècnica de València, Valencia 46022, Spain; Department of Applied Statistics, Operations Research and Quality, Universitat Politècnica de València, Valencia 46022, Spain

## Abstract

**Motivation:**

Deciphering phenotype-specific regulatory mechanisms is key to understanding the molecular basis of complex diseases and traits. However, constructing multi-omic regulatory networks (MO-RNs) is challenging, as it requires integrating heterogeneous omics data, incorporating biological context, and detecting regulatory mechanisms that vary across conditions. The R package MORE (Multi-Omics REgulation) addresses these challenges by applying robust statistical models to infer phenotype-specific regulatory networks from multi-omics data. However, the use of MORE typically requires programming expertise, limiting its accessibility to non-specialist users. To democratize access to advanced multi-omics modeling tools, we present MOREshiny, an interactive web application built on Shiny that extends the module of pathway enrichment analysis and automatically guides the choice of statistical methods.

**Results:**

MOREshiny enables users to upload multi-omic data, configure their models, and interpret results through a user-friendly interface-without the need for coding skills. MOREshiny also allows users to download MORE results for their later exploration and study. To demonstrate the utility of MOREshiny, we showcase its functionalities on a multi-omic ovarian cancer dataset to understand regulatory differences between patients who did or did not require chemotherapy.

**Availability and implementation:**

MOREshiny is freely available for download as a dockerized R Shiny package at https://github.com/BiostatOmics/MOREshiny.

## 1 Introduction

Inferring regulatory networks that explain phenotype-specific differences is crucial for understanding complex biological processes. Incorporating different omic layers into such networks provides a more comprehensive view of regulatory mechanisms. However, tools for multi-omic regulatory network (MO-RN) inference remain scarce, lacking the flexibility to deal with any number or type of omics, incorporate prior regulatory knowledge, and generate and compare phenotype-specific networks.

The MORE R package (Aguerralde-Martin *et al.* 2025) overcomes these limitations and infers phenotype-specific MO-RNs by combining regression models with advanced variable selection techniques while optionally incorporating potential regulatory associations. MORE also provides functionalities for biological interpretation of the inferred regulations.

Although it is a well-documented R package, the use of MORE may be limited to researchers with statistical expertise and R programming skills, which could hinder its adoption by broader biomedical research communities. To address this potential issue, we developed MOREshiny, a user-friendly, interactive Shiny application that extends MORE’s functionalities and makes them accessible to users with limited programming experience. MOREshiny is distributed as a Docker container (Merkel 2014), enabling deployment within a fully configured environment that eliminates the need for manual dependency management or system configuration—challenges often encountered by non-expert users. In addition, MOREshiny incorporates built-in decision-making capabilities to automatically select methodological options based on the input data. This reduces the burden on users to make complex statistical decisions during model construction, further enhancing accessibility for non-specialists.

## 2 Implementation

The MOREshiny interactive application, freely available on GitHub (www.github.com/BiostatOmics/MOREshiny), provides a graphical interface for the MORE tool to infer MO-RNs. This R Shiny package is containerized using a Docker container, ensuring that all necessary dependencies are included and eliminating compatibility issues. Detailed installation instructions for MOREshiny are provided in the Supplementary Note 1, available as supplementary material at *Bioinformatics Advances* online.

**Figure 1:**
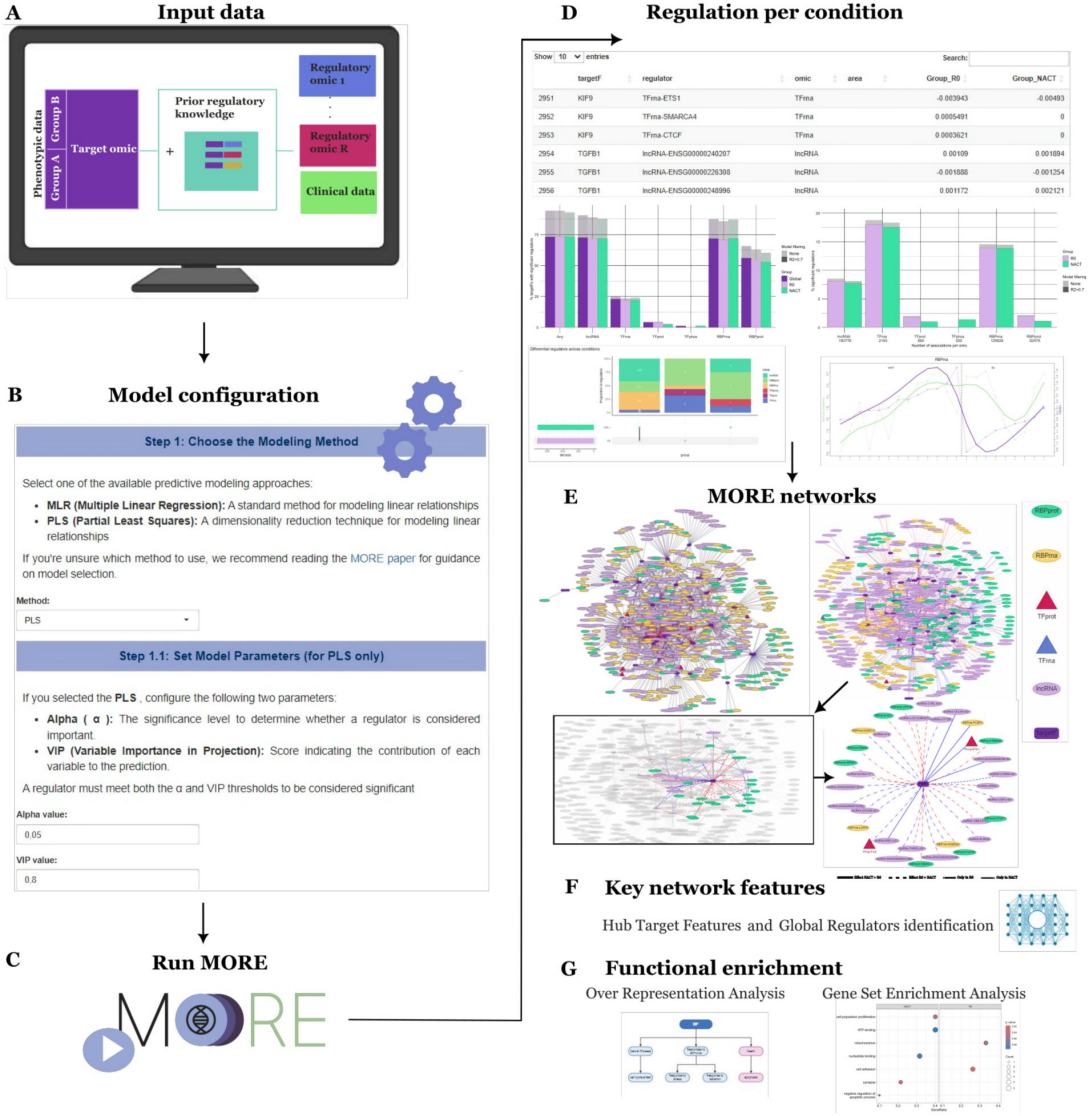
Overview of the MOREshiny application. (A) MOREshiny takes as input a target or “regulated” omic (e.g. gene expression), the regulatory omics (e.g. miRNA expression, DNA methylation, etc.) or clinical data, and the phenotypic groups to be compared (e.g. healthy/disease). Optionally, potential regulatory relationships can be provided (e.g. miRNA target genes). (B) After data loading, the model setting can be configured to apply different regression models and advanced variable selection techniques. (C) The MORE tool is invoked to obtain phenotype-specific MO-RNs. (D) MOREshiny displays a table with the significant regulations per phenotype, which are summarized in different plots, including the number or percentage of regulations per omic and phenotype, common and specific regulations between phenotypes, or regulator-gene profiles. (E) Graphical representation of MO-RNs with activation (blue) and repression (red) regulatory relationships, for the R0 and NACT phenotypes (upper plots) and the differential network analysis for a specific gene (lower plots). (F) MOREshiny allows the identification of hub target features and global regulators. (G) Functional enrichment analysis is provided in MOREshiny with two options: Over Representation Analysis on sets of regulated features sharing the same regulation or Gene Set Enrichment Analysis of regulations per gene or differential regulations between phenotypes.

The MOREshiny user-friendly interface is organized into multiple tabs, each corresponding to a specific step of the analysis workflow, thereby facilitating intuitive navigation and streamlined data exploration. An overview of the tool’s features is presented in Figure 1 and the different steps are next described. In addition, to better guide users through the installation and application of MOREshiny, we provide video and HTML tutorials both within the tool and in the corresponding GitHub repository.

*(A) Input data*. The application requires a data matrix for the target omic, along with one or more data matrices for the corresponding potential regulatory omics, without restrictions on the number or type of these matrices. Input files must have omic features in rows and observations (samples, patients, etc.) in columns, and can be uploaded in *.xlsx*, *.txt*, or *.csv* formats. Optionally, users may include clinical data, prior regulatory knowledge specifying known associations between regulatory and target omic features to guide the network inference process, and phenotype or condition information for the observations if phenotype-specific MO-RNs are to be created; otherwise, all samples are treated as belonging to the same phenotype. Importantly, before proceeding with the analysis, MOREshiny checks that all submitted matrices have the same number of observations, in the same order and with the same labels.

*(B) Model configuration.* Users can interactively select among the different MO-GRN models included in MOREshiny following efficiency comparison in (Aguerralde-Martin *et al.* 2025) (see Supplementary Note 2, available as supplementary material at *Bioinformatics Advances* online), and configure them to infer the MO-RNs. First, users specify whether the regulatory omic data are continuous or binary, and can simultaneously apply an optional filter to exclude regulatory features with low variability across conditions or phenotypes. Second, the user can choose between two regression models: Multiple Linear Regression (MLR) or Partial Least Squares (PLS1). For selecting significant regulations, MLR relies on ElasticNet variable selection combined with a strategy to mitigate the multicollinearity issue (Aguerralde-Martin *et al.* 2025). In PLS, the variable selection method is automatically configured within MOREshiny based on sample size, and users only need to set the significance level and Variable Importance in Projection (VIP) threshold. For model selection guidelines, please see Supplementary Note 2, available as supplementary material at *Bioinformatics Advances* online.

*(C) Running MORE.* The runtime of the analysis depends on the complexity of the model, including the number of samples and features. Upon completion, the results can be downloaded in *.RData* format, allowing future reuse and further exploration in subsequent sessions.

*(D) Regulation per condition.* MOREshiny generates a table listing all significant regulations for each condition, including the corresponding model coefficients, thereby highlighting the relationships between significant regulators and the target features they regulate. In addition, MOREshiny provides a comprehensive suite of plots to assist in the biological interpretation of the inferred networks. Summary plots include the proportion of target omic features with significant regulators and the percentage of significant regulations. In each of these plots, statistics are presented for each regulatory omic and globally as well as per condition or phenotype under comparison. A complementary summary visualization is also provided in the form of an Upset plot which displays differential regulations across conditions. Furthermore, results can be filtered based on model performance metrics such as the explained variance ($R^{2}$) for each target feature, and all displayed outputs are updated accordingly. Additional exploratory visualizations allow users to examine the profiles of specific target features and their significant regulators.

*(E) MORE networks.* MOREshiny generates a customizable network visualization (see Supplementary Note 3, available as supplementary material at *Bioinformatics Advances* online). Users can display the full regulatory network for each phenotype, extract the regulatory network of a specific target feature, or visualize the network for a given pathway. Moreover, the differential network between two phenotypes can be examined for any of these cases. Finally, a filter can also be applied to show only the top X% strongest regulations in the network.

*(F) Key network features.* Information is also provided about relevant features in the network (see Supplementary Note 3, available as supplementary material at *Bioinformatics Advances* online), such as target features with many significant regulations (hub target features) and regulatory omic features that significantly regulate many target features (global regulators).

*(G) Functional enrichment.* Two enrichment approaches were implemented: Over Representation Analysis (ORA) to identify pathways enriched among target features sharing the same significant regulatory feature or omic, and Gene Set Enrichment Analysis (GSEA) to assess the functional implication of highly or differentially regulated target features.

## 3 Use-case: HGSOC multi-omic dataset

We illustrate the usage of MOREshiny on a High-Grade Serous Ovarian Cancer (HGSOC) dataset (Lee *et al.*2020). We generated MO-RNs to compare gene expression regulation between patients who required neoadjuvant chemotherapy (NACT, *n* = 9) and those with complete resection (R0, *n* = 13). The input data integrated transcriptomic, proteomic, and phosphoproteomic layers. Specifically, we used 380 differentially expressed genes (p<0.01, |logFC|>1) as targets, while regulators were transcription factors (TFs), RNA binding proteins, (RBPs), and long non-coding RNAs (lncRNAs), which were selected from annotation databases. Prior knowledge from TFLink was incorporated to focus on documented TF-target interactions (see Supplementary Note 4, available as supplementary material at *Bioinformatics Advances* online). Significant regulations were inferred using a PLS model, with a statistical significance level of 0.01 and a VIP threshold of 1.2.

A total of 38 618 significant regulations were identified. When filtering out models with low goodness of fit ($R^{2}<0.7$), MOREshiny retained 36 842 significant regulations whose proportions across the different regulatory omic layers and phenotypes are displayed in Fig. 1D and Fig. S1, and discussed in Supplementary Note 5, available as supplementary material at *Bioinformatics Advances* online.

Regarding the MO-RNs inferred for R0 and NACT phenotypes, we focused on the *TGFB1* gene to illustrate MOREshiny functionalities (Fig. 1E, Fig. S2, and Supplementary Note 5, available as supplementary material at *Bioinformatics Advances* online). This gene encodes the Transforming Growth Factor Beta 1, whose overexpression is associated with tumor progression, metastasis, and chemoresistance in HGSOC (Wang *et al.* 2018). MOREshiny differential network identified several regulators with known roles in HGSOC biology –such as *NORAD*, *PUF60*, *ETS1*, and *FOS* (Mahner *et al.* 2008, Tomar *et al.* 2018, Zhang *et al.* 2024)– with differential effects across the two phenotypes. Furthermore, potential novel regulators such as *RBM8A* and *SAMD4A* were identified, which are exclusively present in patients with NACT treatment, and have been implicated in the response to neoadjuvant chemotherapy in other cancers (Wang *et al.* 2014, Fei *et al.* 2021), although not yet characterized in the context of HGSOC.

To further validate the MOREshiny functionalities and functional relevance of these findings, we performed an ORA enrichment on the targets of *SAMD4A* and *NORAD* in the NACT cohort (Fig. S3, available as supplementary material at *Bioinformatics Advances* online). For the RNA binding protein *SAMD4A*, enriched pathways included processes critical for tumoral progression, such as positive regulation of apoptotic process, sprouting angiogenesis or cell cycle G1/S transition. In the case of the lncRNA *NORAD*, the ORA identified a highly significant enrichment in the cellular response to TGF-β stimulus, with NORAD regulating 5 out of 6 genes in this pathway. This not only provides a strong functional validation of the *NORAD-TGFB1* regulatory axis identified by MOREshiny but also brings to light these regulators as potential prognostic biomarkers and therapeutic targets for overcoming chemoresistance in HGSOC (Capela *et al.* 2024).

## 4 Discussion

MOREshiny is a user-friendly Shiny interface designed to simplify the inference of MO-RNs. While the underlying MORE framework offers powerful capabilities, its reliance on programming and statistical knowledge can limit accessibility. MOREshiny overcomes this by providing an intuitive graphical interface and by extending the functionalities of pathway enrichment analysis on the resulting regulatory networks. Dockerization further enhances usability by enabling easy deployment with minimal setup.

Naturally, some limitations arise for complex studies where the number of regulations to be analysed requires intensive computation. In that case, we recommend the MORE R package instead, as it supports parallelisation and improved visualization for large regulatory networks with Cytoscape. An alternative visualization option in MOREshiny would be reducing the number of regulations in the network, which is facilitated by the filtering options provided in the tool.

Consistent with the MORE framework, the identified relationships by MOREshiny represent statistical inferences of regulation. While our previous benchmarks demonstrate high accuracy in simulated environments, these associations should be viewed as high-confidence candidates for subsequent experimental validation.

This work illustrates how MOREshiny contributes to the democratization of advanced computational tools for modelling regulatory mechanisms in complex biological systems.

## Supplementary Material

vbag175_Supplementary_Data

## Availability and implementation

The MOREshiny Shiny package is freely available at https://github.com/BiostatOmics/MOREshiny under the GNU Public License. The HGSOC data used in this work were downloaded from the European Genome-Phenome Archive (EGA) under accession number EGAS00001003804.
