# Supplementary material for: MOREshiny: a user-friendly application for the inference of phenotype-specific multi-omic regulatory networks
Source: Bioinform Adv. 2026 Jun 18;6(1):vbag175. doi: 10.1093/bioadv/vbag175 (PMC13310010; doi:10.1093/bioadv/vbag175)
Supplement: vbag175_Supplementary_Data [file vbag175_supplementary_data.pdf]

Supplementary Data
MOREshiny: a user-friendly application for the inference of phenotype-specific multi-omic regulatory networks

Maider Aguerralde-Martin,[1] Roxana Andreea Moldovan,[1,2] María Verdú[1] and Sonia Tarazona[1,*]

[1]Department of Applied Statistics, Operations Research and Quality, Universitat Politècnica de València, Valencia, 46022, Spain and
[2]Institute for Integrative Systems Biology, Spanish National Research Council, Catedràtic Agustín Escardino Benlloch, Paterna, 46980, Spain
[*]Corresponding author. sotacam@eio.upv.es

Supplementary Note 1: MOREshiny installation

As MOREshiny runs in a Docker container, users must have Docker installed on their system before installing and using MOREshiny. Once MOREshiny has been downloaded, it can be launched in three simple steps:

1. Open a command-line terminal in the folder where MOREshiny was downloaded, and build the Docker image by running: *docker build -t moreshiny .*
2. Once the build is complete, start the application with: *docker run -p 8180:8180 moreshiny*
3. Open a web browser and navigate to *http://localhost:8180/* to access the application

Internet connection is required only for the initial setup. In subsequent sessions, the application can be relaunched by repeating steps 2 and 3, or by directly starting the previously created Docker image via the Docker environment.

Supplementary Note 2: Recommendations for model selection

The MOREshiny application implements the MORE methodology; comprehensive details regarding the underlying statistical models, performance benchmarks, and data specifications are documented in Aguerralde-Martin et al. [1]. However, to assist users in their analysis, we provide the following core specifications and guidelines:

Selection of Target and Regulatory Omics

To construct multi-omic regulatory networks (MO-RN) with MORE, users must define the biological roles of their data layers based on the direction of hypothesized regulation. The target omic contains the features whose regulation is to be studied — typically downstream molecular layers like gene expression or protein levels where phenotypic differences are observed. Conversely, regulatory omics provide the "potential regulators" or upstream drivers of the target features. In the case of gene expression, such regulators could be transcription factors (TFs), microRNAs (miRNA), DNA methylation, or Copy Number Variations (CNVs), among others. The tool offers complete flexibility to include any omic modality as a regulatory omic, so users must decide the target omic and the potential regulatory layers. It is important to note that the statistical performance of the underlying MORE algorithm depends on the number of available observations. While accuracy degrades as the number of samples decreases, previous benchmarking has shown that results remain reliable with at least five samples per condition [1]. Naturally, including large numbers of regulators with small sample sizes could compromise the robustness of the results.

Data Preprocessing and Parameters

Standardized data preprocessing, including robust normalization and the filtering of low-count features, is recommended when using MOREshiny. While these initial steps should be performed before data upload, the application includes built-in functionalities to further refine the dataset. Specifically, it is strongly recommended to apply the MOREshiny low-variability filter to exclude features that lack sufficient variance to act as potential regulators across the biological conditions under study.

Furthermore, when the objective is to infer phenotype-specific MO-RN networks, the analysis should be restricted to target omic features that are differentially expressed across the phenotypes under study. Focusing on such differentially expressed features ensures that the resulting MO-RN captures meaningful regulatory disparities rather than baseline cellular activity.

© The Author . Published by Oxford University Press. All rights reserved. For permissions, please e-mail: journals.permissions@oup.com

Statistical Frameworks

The MOREshiny graphical interface allows users to select between the Multiple Linear Regression (MLR) and Partial Least Squares (PLS) approaches implemented within the MORE framework. The selection between them should be done depending on the statistical properties of the users data and the desired biological interpretation. While MLR is a standard choice for model construction, PLS is specifically recommended when the target omic data is not normally distributed, as it does not rely on any distributional assumptions. Furthermore, PLS is the required option if the uploaded datasets contain missing values, as the underlying NIPALS algorithm can effectively handle incomplete data. In contrast, MLR methods will exclude any observations containing missing values.

Model benchmarking in [1] guided the choice of variable selection procedures within each regression method in MOREshiny. For MLR, ISGL was not implemented in MOREshiny due to its poorer trade-off between performance and computational efficiency, whereas MOREshiny automatically selects Jackknife or permutation strategies in PLS1 based on the available sample size. PLS2 was neither implemented, as it requires more computational resources than its counterpart PLS1 and advanced statistical knowledge for interpretation.

The user should also understand that MLR and PLS models differ fundamentally in how they handle comparisons between phenotypic conditions and therefore the interpretation of the results. MLR takes one of the conditions as a reference and compares the remaining groups against it; consequently, the regression coefficients in the resulting 'Regulation Per Condition´ represent the expected change in the target omic feature when transitioning from the reference group to the phenotype under study, incorporating the interaction between the specific regulator and the condition where applicable. In contrast, PLS regression coefficients reflect the expected change relative to the population mean for an observation belonging to a specific group, while accounting for relevant regulatory interactions when applicable.

PLS Model Parameterization

For PLS models, users must specify both the significance level (α) and the Variable Importance in Projection (VIP) threshold to identify significant regulatory relationships. A regulator is considered significant only if both conditions are met simultaneously. The α value represents the significance level for the regression coefficients, calculated using either a Jackknife or a permutation technique.

A lower α (e.g., 0.01 instead of 0.05) increases the statistical stringency required to reject the null hypothesis. Complementarily, the VIP score quantifies each predictor's contribution to the model; a higher VIP score indicates that a regulator has greater explanatory power. While a default $VIP > 0.8$ and $\alpha = 0.05$ are recommended for initial exploratory analyses, users should adopt stricter values (higher VIP and lower α) when the priority is to minimize false positives and identify only the most robust regulatory drivers.

Computational cost

For reference, executing the PLS model on the example dataset required 3 minutes and 45 seconds on a standard laptop (13th Gen Intel Core i7-1360P, 2.20 GHz, 32 GB RAM, Windows 11). Please note that total computation time scales with the number of target and regulatory omic features included in the analysis.

Supplementary Note 3: MORE networks

Network visualization

MOREshiny provides its own customizable network visualization. While the original R MORE package generates the network visualization with *igraph* [3, 8] (with more limited options) or externally with *Cytoscape* [23], MOREshiny significantly simplifies this task by having the visualization embedded in the tool. For that, we used the *visNetwork* R package [2]. In addition, MOREshiny users can choose to connect omic features with arrows indicating regulatory direction or with simple lines indicating coexpression.

While the R package can automatically plot either the whole network for a specific phenotype or condition or the subnetwork corresponding to a given pathway, MOREshiny also allows users to select a target feature to visualize its corresponding subnetwork. The same applies to differential networks.

Key network features

MOREshiny provides a list of key features for a given network. We considered two types of key features:

- **Hub target features.** A target feature is defined as "hub" when having many significant regulators, specifically, when the number of regulators is no fewer than 10 and having a network degree exceeding the third quartile of all network degrees.
- **Global regulators.** A regulator is considered to be a "global regulator" when regulating no fewer than 10 target features and having a network degree exceeding the third quartile of all network degrees.

These key features are relevant to defining the different types of pathway enrichment analysis implemented in MOREshiny, as described next.

Functional enrichment

Each type of pathway enrichment analysis in MOREshiny offers different approaches for identifying pathways significantly altered by the studied regulations. Users can provide their own annotation file but, to facilitate this analysis for non-expert users, we have also incorporated the Gene Ontology annotation database for human and mouse into the app.

The *Over Representation Analysis* (ORA) focuses on a specific phenotype or condition and returns pathways that are enriched among (1) hub target features, (2) target features significantly regulated by the same global regulator, or (3) target features significantly regulated by a given regulatory omic (e.g. methylation). When choosing option (2), MOREshiny users can select the global regulator of interest.

The *Gene Set Enrichment Analysis* (GSEA) can be applied to a specific phenotype or condition or to compare two phenotypes or conditions. For only one condition, the target features are ranked by the number of significant regulators. For two given conditions, the ranking is based on a score that compares the number of significant regulators between them. Therefore, the pathways returned by MOREshiny are those enriched among highly regulated target features or with different regulation between conditions, depending on the choice.

In MOREshiny, we have implemented plots to graphically summarize both ORA and GSEA results (see an example in Figure S3).

Supplementary Note 4: Data acquisition and preprocessing

A high-grade serous ovarian cancer (HGSOC) dataset from [15] was used to demonstrate the functionalities of MOREshiny. The HGSOC dataset consists of transcriptomics, proteomic and phosphoproteomic data measured on 22 patients, comprising 9 who received neoadjuvant chemotherapy (NACT) after complete gross resection, and 13 who did not receive chemotherapy (R0).

Transcriptomic data generated by RNA-seq were downloaded from the European Genome-Phenome Archive (EGA; https://ega-archive.org/) [13] under accession number EGAS00001003804, provided in BAM format. Gene-level quantification was performed using the featureCounts tool [16]. The resulting raw counts underwent quality control with the NOISeq package [26, 27], followed by conditional quantile normalisation with the cqn package [12]. Differential expression analysis was then performed with limma [24], applying a p-value threshold of 0.01 and an absolute log fold change greater than 1. This analysis identified 380 differentially expressed genes, which were subsequently used as target genes in MOREshiny.

In addition, transcriptomic data was used to derive three regulatory omic layers: long non-coding RNAs (lncRNA), RNA-binding proteins (RBPrna), and transcription factors (TFrna). The 462 lncRNAs were identified using ENSEMBL gene annotations retrieved via the biomaRt package [9]. The 361 RNA-binding proteins (RBP) were annotated from the RBPDB database [7]. The 1,297 transcription factors (TF) were retrieved from the TFLink database [17]. Gene expression quantification for all these features was obtained from the transcriptomic data.

Proteomic and phosphoproteomic data were obtained from the supplementary materials of the original publication [15]. The proteomic dataset was already normalised and did not require further processing. From this dataset, we derived two additional regulatory layers: RBP proteomic quantification (RBPprot) for 237 RBPs and TF proteomic quantification (TFprot) for 636 TFs. Phosphoproteomic data preprocessing included missing value imputation. Only phosphorylation sites detected in at least one sample per condition and in at least 70% of all samples were retained. Missing values were imputed using the *slsa* algorithm from the imp4p package [11], and the data were subsequently normalised using cyclic loess normalisation as implemented in the limma package [24]. Quality control was performed before and after imputation and normalisation steps. The processed phosphoproteomic data rendered an additional regulatory layer: phosphorilation of 181 TFs (TFphos).

Prior regulatory knowledge was only incorporated for TFs. Specifically, TF–target gene interactions were retrieved from the TFLink database and considered for TFs derived from transcriptomic (TFrna), proteomic (TFprot), and phosphoproteomic data (TFphos).

Supplementary Note 5: MOREshiny HGSOC results

To investigate the differential molecular mechanisms underlying patients who underwent RO and NACT treatments, we uploaded the multi-omic datasets described in Supplementary Note 4 to MOREshiny. Based on this input, MOREshiny identified significant regulatory interactions involving transcription factors (TFs), RNA-binding proteins (RBPs), and long non-coding RNAs (lncRNAs). Out of the initial 380 target genes, 330 were found to be significantly regulated by at least one of these regulatory layers. In total, MOREshiny identified 38,618 significant regulations, 36,842 of which corresponded to 256 genes with an $R^2 > 0.7$—genes with better explained regulations.

The percentage of genes with significant regulators varied by omic modality, although similar percentages were observed across treatments (Fig. S1A). Genes were predominantly regulated by lncRNAs (around 73%), closely followed by RBPs—either inferred from RNA-seq (RBPrna) or proteomics (RBPprot). In contrast, fewer than 25% of genes were significantly regulated by TFs, with phosphoproteomics-derived TFs (TFphos) showing virtually no significant regulatory associations. This disparity highlights the varying regulatory influence exerted by each omic layer and underscores the complexity of post-transcriptional and transcriptional regulation in the dataset.

Considering the initial regulatory associations provided to MOREshiny (Fig. S1B), TFs had the fewest initial associations, with 2,193 TFrna, 890 TFprot, and 290 TFphos associations. However, TFrna exhibited the highest percentage of significant regulations ($> 17\%$), followed by RBPrna ($> 14\%$). Across all omic types—except for TFphos—the proportion of significant regulations tended to decrease in patients who underwent the more aggressive, NACT, treatment.

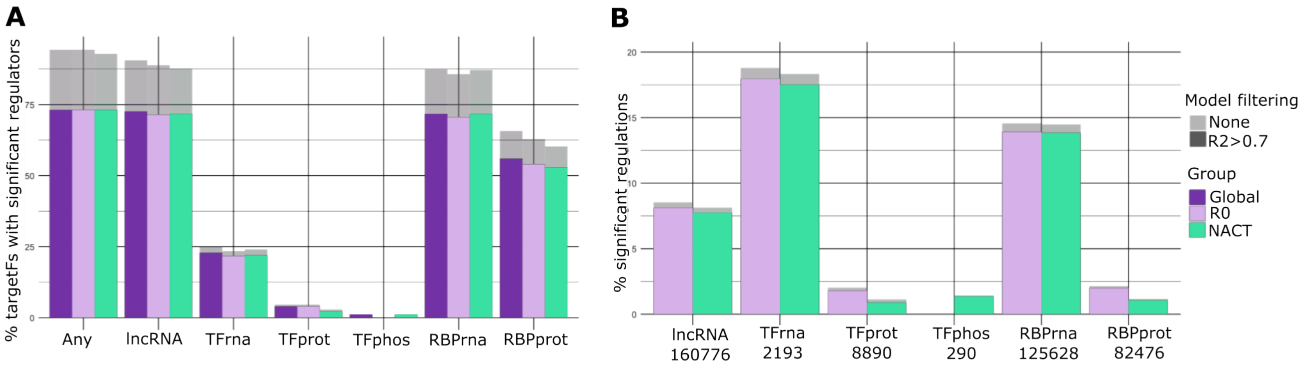

Fig. S1. Summary of significant regulations identified by PLS models on the ovarian cancer data and extracted via MOREshiny. Transparent colours correspond to results derived from all fitted models. Solid colours correspond to models with $R^2 > 0.7$. A) The first set of bars shows the percentage of genes with significant regulations by any omic, globally or per treatment. The following sets of bars refer to each omic modality and show the percentage of genes with significant regulations for that omic, globally or per treatment. B) The percentage of significant regulations per omic over the initial potential regulations (associations) is displayed for each treatment.

Among the 256 genes with better-explained regulations, several have previously been implicated in the pathogenesis of HGSOC, including *RUNX1*, *TGFBI*, *ID4*, and *EIF5A2* [6, 14, 20, 22]. Notably, we highlight *TGFB1*, which encodes the Transforming Growth Factor Beta 1, a key protein involved in development, cell proliferation, homeostasis, immune response, and apoptosis. The TGFB1 signalling pathway has been linked to various cancers, including HGSOC, where its overexpression is associated with tumour progression, metastasis, and chemoresistance [30]. Given its relevance, *TGFB1* has been proposed as both a biomarker and a potential therapeutic target in this cancer type [33].

Consistent with these observations, in our study, *TGFB1* expression was increased in patients treated with NACT compared to those in the R0 group. To further explore this difference, we examined the differential regulatory network of *TGFB1* between the two treatment groups (Fig. S2). For the ease of the network visualisation, in Fig. S2 we represent the 50% of the regulations with the strongest associations with the expression of *TGFB1*. Among these differential regulations, we identified several regulators with established roles in HGSOC biology, including *NORAD*, *PUF60*, *ETS1*, and *FOS*.

NORAD is a long non-coding RNA whose overexpression has been associated with increased tumour cell migration and invasion [18, 31]. *PUF60* is an RNA-binding protein involved in alternative splicing and transcriptional regulation, and its activation promotes the proliferation of ovarian epithelial tumour cells [34]. The transcription factors *ETS1* and *FOS* have been linked to metastasis and disease progression in ovarian carcinoma, with prognostic implications [21, 28].

Some of these regulators have already been described as modulators of *TGFB1* in other oncological contexts, while others may represent novel regulatory interactions. For example, in vitro studies in breast cancer models have shown that silencing *NORAD* reduces *TGFB1* expression and pathway activation, leading to decreased tumour proliferation and improved patient survival [4]. In our analysis, *NORAD* was identified as a regulator of *TGFB1* exclusively in the NACT group, which may contribute to treatment resistance and treatment response in these patients. In prostate tumours, high levels of *ETS1* have been shown to induce TGFB signalling by increasing *TGFB1* expression [25]. In our study, this regulation was observed in both groups, although it had a higher expression in the NACT group, which could be associated with their poorer prognosis.

Additionally, we identified regulators previously associated with treatment response in other tumour contexts but not previously characterised in HGSOC. For instance, *RBM8A*, an RNA-binding protein involved in cell growth, metastasis, and apoptosis, has been shown to enhance cisplatin resistance and cell proliferation in breast cancer [29]. Similarly, *SAMD4A*, implicated in neoadjuvant chemoresistance in pancreatic adenocarcinoma [10], directly participates in TGFB1 signalling and has been reported to engage in positive feedback regulation of *TGFB1* expression [19, 35]. In our dataset, *RBM8A* and *SAMD4A* were found only in patients who received NACT, highlighting their essential role in chemotherapy response and resistance.

In order to elucidate the functional impact of these regulators in the NACT cohort, an Over-Representation Analysis (ORA) was performed on the regulatory targets in this condition. The analyses were conducted using the Gene Ontology (GO) Biological Processes database via the org.Hs.eg.db R package [5], applying a significance threshold of $p < 0.05$. For further discussion we focus on the regulatory targets of the RBP *SAMD4A* and the lncRNA *NORAD* (Fig. S3).

The ORA identified 9 significantly enriched biological processes for *SAMD4A*, primarily related to the apoptotic process, cell growth and differentiation, cell cycle regulation and sprouting angiogenesis (Fig. S3A). These results suggest that *SAMD4A* may play a role in coordinating a complex response involving both cell turnover and the modulation of the tumour microenvironment in patients undergoing chemotherapy. In the context of gynaecological cancers, including HGSOC, high microvessel density has been frequently associated with poor prognosis and a higher risk of recurrence [32]. Consequently, the role of *SAMD4A* in angiogenesis could facilitate the restoration of nutrient supply to surviving tumor cells, which may contribute to the clinical outcomes of NACT group.

In the case of lncRNA *NORAD*, the analysis revealed 23 significant biological processes. The most relevant enrichment was the cellular response to TGF-β stimulus, where *NORAD* was found to regulate 5 out of 6 genes associated with this term. This finding provides a robust functional validation of our previous results, which identified NORAD as a specific regulator of *TGFB1* exclusively in the NACT group. Furthermore, *NORAD* targets were found to be enriched in terms related to the positive regulation

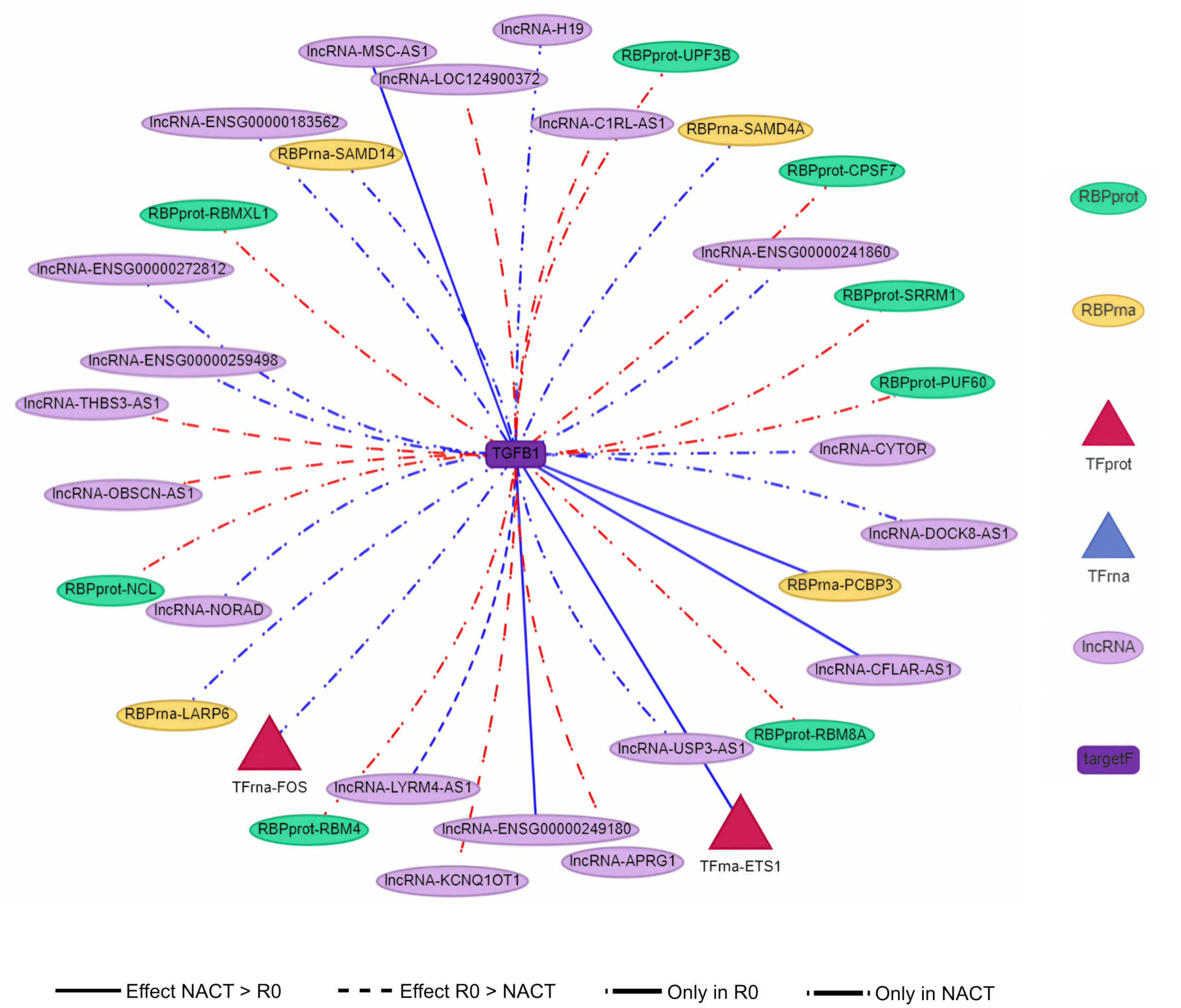

Fig. S2. Differential network between R0 and NACT extracted from MOREshiny for the ovarian cancer data. In the network, node colours indicate the omic modalities: green for RNA-binding proteins extracted from proteomics (RBPprot), yellow for RNA-binding proteins extracted from RNA-seq (RBPrna), red for transcription factors extracted from RNA-seq (TFrna), light purple for long non-coding RNAs (lncRNA) and purple for genes. Straight lines represent a stronger regulatory effect in the NACT treatment compared to the R0. Dashed lines represent a stronger regulatory effect in the R0 compared to the NACT treatment. Dot dashed lines represent a regulatory effect only significant for the R0. Dot-dash-dot lines represent a regulatory effect only significant for the NACT.

of apoptotic processes, G1/S transition, regulation of cyclin-dependent protein kinase activity, and negative regulation of epithelial cell differentiation (Fig. S3B). These processes are closely linked to survival and patient prognosis in HGSOC [36], confirming the central role of NORAD in the NACT-specific regulatory landscape. Our findings indicate that NORAD has the potential to function as a prognostic biomarker and therapeutic target in ovarian cancer, thereby extending its established role in breast cancer survival [4] to the context of advanced HGSOC.

However, a more detailed stratification of NACT patients based on their clinical outcomes would allow a refined analysis of the roles of these regulators in chemoresistance, and further research is needed to validate these regulatory mechanisms and their implications in treatment response.

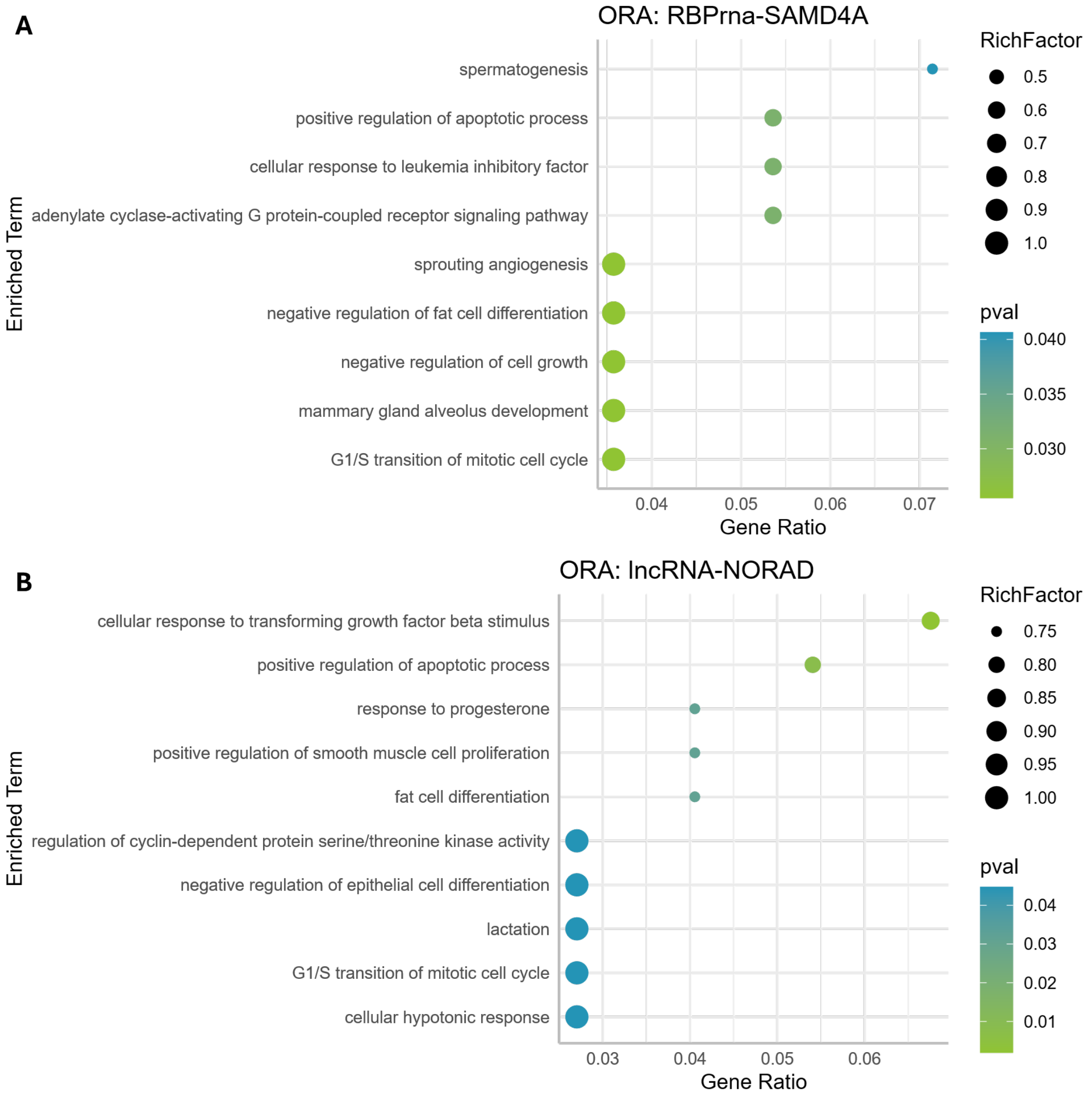

Fig. S3. Over-representation analysis results for *SAMD4A* and *NORAD* regulatory targets in the NACT condition. Dotplots illustrate the top enriched GO biological processes for the regulatory targets of (A) RBPrna-SAMD4A and (B) the long non-coding RNA lncRNA-NORAD. The x-axis represents the proportion of target genes that belong to a given GO term, as listed in the y-axis. The size of each dot corresponds to the Rich Factor, calculated as the ratio of target genes to the total number of genes in the GO term, and the colour gradient indicates the statistical significance based on the p-value, establishing a significance threshold of $p < 0.05$.

References

Aguerralde-Martin M, Clemente-Císcar M, Conesa A *et al.* More interpretable multi-omic regulatory networks to characterise phenotypes. *Briefings in Bioinformatics* 2025;26:bbaf270.

Almende B.V. and Contributors, Thieurmel B (2025) *visNetwork: Network Visualization using 'vis.js' Library.* R package version 2.1.4.

Antonov M, Csárdi G, Horvát S *et al.* igraph enables fast and robust network analysis across programming languages. *arXiv preprint arXiv:2311.10260* 2023.

Capela AM, Tavares-Marcos C, Estima-Arede HF *et al.* Norad-regulated signaling pathways in breast cancer progression. *Cancers* 2024;16:636.

Carlson M, Falcon S, Pages H *et al.* org. hs. eg. db: genome wide annotation for human. *R package version* 2019;3:3.

Chen Y, He Z, Yang S *et al.* Runx1 knockdown induced apoptosis and impaired emt in high-grade serous ovarian cancer cells. *Journal of Translational Medicine* 2023;21:886.

Cook KB, Kazan H, Zuberi K *et al.* Rbpdb: a database of rna-binding specificities. *Nucleic acids research* 2010;39:D301–D308.

Csárdi G, Nepusz T. The igraph software package for complex network research. *InterJournal* 2006;Complex Systems:1695.

Durinck S, Spellman PT, Birney E *et al.* Mapping identifiers for the integration of genomic datasets with the r/bioconductor package biomart. *Nature Protocols* 2009;4:1184–1191.

Fei N, Wen S, Ramanathan R *et al.* Smad4 loss is associated with response to neoadjuvant chemotherapy plus hydroxychloroquine in patients with pancreatic adenocarcinoma. *Clinical and translational science* 2021;14:1822–1829.

Gianetto QG (2021) *imp4p: Imputation for Proteomics*. R package version 1.2.

Hansen KD, Irizarry RA, Wu Z. Removing technical variability in RNA-seq data using conditional quantile normalization. *Biostatistics* 2012;13:204–216.

Lappalainen I, Almeida-King J, Kumanduri V *et al.* The european genome-phenome archive of human data consented for biomedical research. *Nature Genetics* 2015;47:692–695.

Lecker LS, Berlato C, Maniati E *et al.* Tgfbi production by macrophages contributes to an immunosuppressive microenvironment in ovarian cancer. *Cancer research* 2021;81:5706–5719.

Lee S, Zhao L, Rojas C *et al.* Molecular analysis of clinically defined subsets of high-grade serous ovarian cancer. *Cell reports* 2020;31.

Liao Y, Smyth GK, Shi W. featurecounts: an efficient general purpose program for assigning sequence reads to genomic features. *Bioinformatics* 2014;30:923–930.

Liska O, Bohár B, Hidas A *et al.* Tflink: an integrated gateway to access transcription factor–target gene interactions for multiple species. *Database* 2022;2022:baac083.

Liu L, Guo J, Pang Xl *et al.* Exploration of the mechanism of norad activation of tgf-β1/smad3 through mir-136-5p and promotion of tacrolimus-induced renal fibrosis. *Renal Failure* 2023;45:2147083.

Liu L, Li Q, Yang L *et al.* Smad4 feedback activates the canonical tgf-β family signaling pathways. *International journal of molecular sciences* 2021;22:10024.

Lu X, Ji C, Jiang L *et al.* Tumour microenvironment-based molecular profiling reveals ideal candidates for high-grade serous ovarian cancer immunotherapy. *Cell proliferation* 2021;54:e12979.

Mahner S, Baasch C, Schwarz J *et al.* C-fos expression is a molecular predictor of progression and survival in epithelial ovarian carcinoma. *British journal of cancer* 2008;99:1269–1275.

Manasa P, Sidhanth C, Krishnapriya S *et al.* Oncogenes in high grade serous adenocarcinoma of the ovary. *Genes & cancer* 2020;11:122.

P S, A M, O O *et al.* Cytoscape: a software environment for integrated models of biomolecular interaction networks. *Genome Res* 2003;13:2498–2504.

Ritchie ME, Phipson B, Wu D *et al.* limma powers differential expression analyses for RNA-sequencing and microarray studies. *Nucleic Acids Research* 2015;43:e47.

Rodgers JJ, McClure R, Epis MR *et al.* Ets1 induces transforming growth factor β signaling and promotes epithelial-to-mesenchymal transition in prostate cancer cells. *Journal of Cellular Biochemistry* 2019;120:848–860.

Tarazona S, Furio-Tari P, Turra D *et al.* Data quality aware analysis of differential expression in rna-seq with noiseq r/bioc package. *Nucleic Acids Research* 2015;43:e140.

Tarazona S, Garcia-Alcalde F, Dopazo J *et al.* Differential expression in rna-seq: a matter of depth. *Genome Research* 2011;21:4436.

Tomar S, Plotnik JP, Haley J *et al.* Ets1 induction by the microenvironment promotes ovarian cancer metastasis through focal adhesion kinase. *Cancer letters* 2018;414:190–204.

Wang Y, Chen D, Qian H *et al.* The splicing factor rbm4 controls apoptosis, proliferation, and migration to suppress tumor progression. *Cancer cell* 2014;26:374–389.

Wang Y, Xiang J, Wang J *et al.* Downregulation of tgf-β1 suppressed proliferation and increased chemosensitivity of ovarian cancer cells by promoting brca1/smad3 signaling. *Biological Research* 2018;51:1–7.

Xu C, Zhu LX, Sun DM *et al.* Regulatory mechanism of lncrna norad on proliferation and invasion of ovarian cancer cells through mir-199a-3p. *European Review for Medical & Pharmacological Sciences* 2020;24.

Yetkin-Arik B, Kastelein AW, Klaassen I *et al.* Angiogenesis in gynecological cancers and the options for anti-angiogenesis therapy. *Biochimica et Biophysica Acta (BBA)-Reviews on Cancer* 2021;1875:188446.

Yue H, Li W, Chen R *et al.* Stromal postn induced by tgf-β1 facilitates the migration and invasion of ovarian cancer. *Gynecologic oncology* 2021;160:530–538.

Zhang C, Ni X, Tao C *et al.* Targeting puf60 prevents tumor progression by retarding mrna decay of oxidative phosphorylation in ovarian cancer. *Cellular Oncology* 2024;47:157–174.

Zhao M, Mishra L, Deng CX. The role of tgf-β/smad4 signaling in cancer. *International journal of biological sciences* 2018;14:111.

Zhao Y, Yang SM, Jin YL *et al.* A robust gene expression prognostic signature for overall survival in high-grade serous ovarian cancer. *Journal of oncology* 2019;2019:3614207.
